# Up-regulation of serotonin receptor 2B mRNA and protein in the peri-infarcted area of aged rats and stroke patients

**DOI:** 10.18632/oncotarget.8277

**Published:** 2016-03-22

**Authors:** Ana-Maria Buga, Ovidiu Ciobanu, George Mihai Bădescu, Catalin Bogdan, Ria Weston, Mark Slevin, Mario Di Napoli, Aurel Popa-Wagner

**Affiliations:** ^1^ Department of Psychiatry and Psychotheraphy, University of Medicine Rostock, Rostock, Germany; ^2^ Center of Clinical and Experimental Medicine, University of Medicine and Pharmacy Craiova, Craiova, Romania; ^3^ Department of Healthcare Science, Manchester Metropolitan University, Manchester, UK; ^4^ Neurological Service, San Camillo de' Lellis General Hospital, Rieti, Italy; ^5^ Neurological Section, SMDN—Center for Cardiovascular Medicine and Cerebrovascular Disease Prevention, Sulmona, L'Aquila, Italy; ^6^ Vivantes Humboldt-Klinikum, Center for Affective Disorders, Berlin, Germany; ^7^ Psychiatry Clinical Hospital, University of Medicine and Pharmacy of Craiova, Craiova, Romania

**Keywords:** aging, stroke, post-stroke depression, serotonin receptor type B, neurogenesis, Gerotarget

## Abstract

Despite the fact that a high proportion of elderly stroke patients develop mood disorders, the mechanisms underlying late-onset neuropsychiatric and neurocognitive symptoms have so far received little attention in the field of neurobiology. In rodents, aged animals display depressive symptoms following stroke, whereas young animals recover fairly well. This finding has prompted us to investigate the expression of serotonin receptors 2A and 2B, which are directly linked to depression, in the brains of aged and young rats following stroke. Although the development of the infarct was more rapid in aged rats in the first 3 days after stroke, by day 14 the cortical infarcts were similar in size in both age groups i.e. 45% of total cortical volume in young rats and 55.7% in aged rats. We also found that the expression of serotonin receptor type B mRNA was markedly increased in the perilesional area of aged rats as compared to the younger counterparts. Furthermore, histologically, HTR2B protein expression in degenerating neurons was closely associated with activated microglia both in aged rats and human subjects. Treatment with fluoxetine attenuated the expression of Htr2B mRNA, stimulated post-stroke neurogenesis in the subventricular zone and was associated with an improved anhedonic behavior and an increased activity in the forced swim test in aged animals. We hypothesize that HTR2B expression in the infarcted territory may render degenerating neurons susceptible to attack by activated microglia and thus aggravate the consequences of stroke.

## INTRODUCTION

Stroke is the second-leading global cause of death behind heart disease, accounting for 11.13% of total deaths worldwide in 2015 [1]. Studies of stroke have demonstrated age and gender effects on incidence, functional recovery and mortality, not only in humans but also in animal models [2-4]. Therefore, studies on physiologically complex organisms including rats, mice and non-human primates are required to investigate the molecular mechanisms of aging in humans, to predict human responses to age-related diseases and also the response of aged organisms to therapeutic interventions. Since epidemiological studies have revealed that human stroke occurs more often in old ages (more than 80 years) than late middle age (60-79 years) [1], it is advisable to use rats of appropriate age to study the behavioral and molecular mechanisms underlying functional recovery after stroke in animal model. Therefore, the aged rodent model offers a useful tool to investigate the molecular pathways and potential novel therapies that may improve functional outcome after cerebral ischemia in preclinical studies [5].

Post-stroke depression (PSD) is among the most frequent neuropsychiatric consequences of stroke. Depression also negatively influences stroke outcome with increased morbidity, mortality and poorer functional recovery [6, 7]. Morphologically, depression is associated with atrophy and loss of neurons and glia, which contribute to decreased size and function of limbic brain regions that control mood and depression, including the prefrontal cortex and hippocampus [8].

In addition to an increased risk of mortality, depressed patients are more likely to develop coronary artery disease, type 2 diabetes and cognitive deficits [9-12]. Depression also complicates the prognosis of other chronic diseases [13, 14]. However, the biological mechanisms underlying depression remains poorly understood due to a lack of biomarkers, relatively low rates of heritability, and heterogeneity of precipitating factors, including stress [15-17]. For example, a recent study has linked HTR2b polymorphisms to susceptibility to migraine/hypoperfusion without aura in a Spanish population [18].

In the normal adult brain, 5-HT receptor mRNA is present at high concentrations in the frontal cortex, basal ganglia and at lower levels in some components of the limbic system (including hippocampus, septum and amygdala) [19], while the highest expression of 5-HT2C receptor is found in the choroid plexus, with lower but substantial densities in other regions, lateral septal nucleus, subthalamic nucleus, amygdala and parts of the hippocampus, substantia nigra, central gray and cerebellum [20]. However, 5-HT2C did not show an age-dependency in post-stroke rats and was, therefore, not investigate din this study. 5-HT2B receptor mRNA is mainly found in the hippocampus as well as the cortex, midbrain and the hypothalamus [21].

At the genetic and cellular level, we previously found significant differences in behavioral, cytological and genomic responses to injury in aged animals compared to younger controls [22-25]. Behaviorally, aged animals display depressive symptoms following stroke, whereas young animals recover fairly well [26]. This finding has been confirmed repeatedly in subsequent studies [25, 27], prompting us to investigate the expression of serotonin receptors 2A and 2B, which are directly linked to depression, in the brains of aged and young rats following stroke.

## RESULTS

To facilitate feeding in aged animals during the first three days post-stroke, we fed them with moistened, soft pellets. Nevertheless, aged animals do lose some weight in the first week following stroke. The mortality rate was higher for aged rats (33%) than for younger rats (15%) during this time. As previously reported, the hematologic parameters showed some age-associated differences, notably in blood pressure, but the differences were not statistically significant in this stroke model [22, 32].

### Hedonic behavior

**Figure 1:**
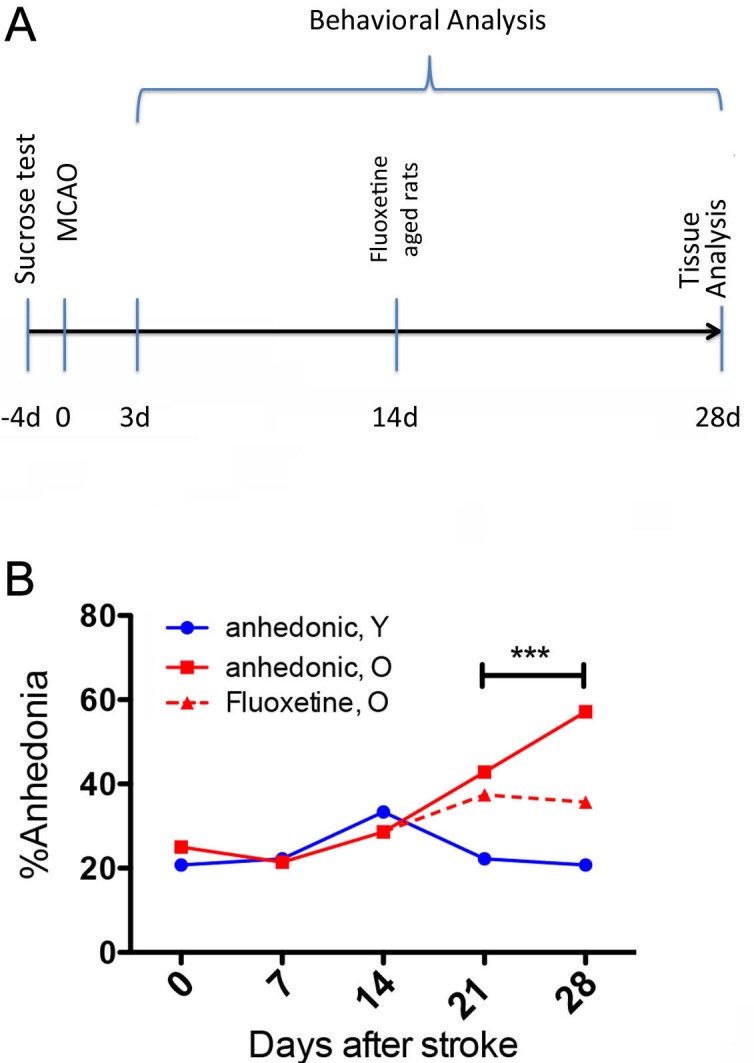
Experimental design and time course of spontaneous activity and hedonic behaviour after stroke **A.** Schematic overview of the experimental design.**B.**: Before surgery, 79% of young animals (blue line) and 75% of old animals (red line) were hedonic. After stroke, the number of anhedonic animals increased to 42% by day 21 and to 57% by day 28. The fluoxetine treatment however did reduce anhedonic behaviour to 35% by day 28 (Figure 1B, red dashed line). ** *p* < .020.

Before surgery, 79% of young animals and 75% of old animals were hedonic (Figure 1B). After stroke, the number of hedonic young animals did not change significantly over the 28 days testing period. However, in the old group the number of anhedonic animals has increased to 42% by day 21 and to 57% by day 28 (Figure 1B). The fluoxetine treatment however has reduced anhedonic behaviour to 35% by day 28 (Figure 1B, dashed line).

### Forced swim

**Figure 2:**
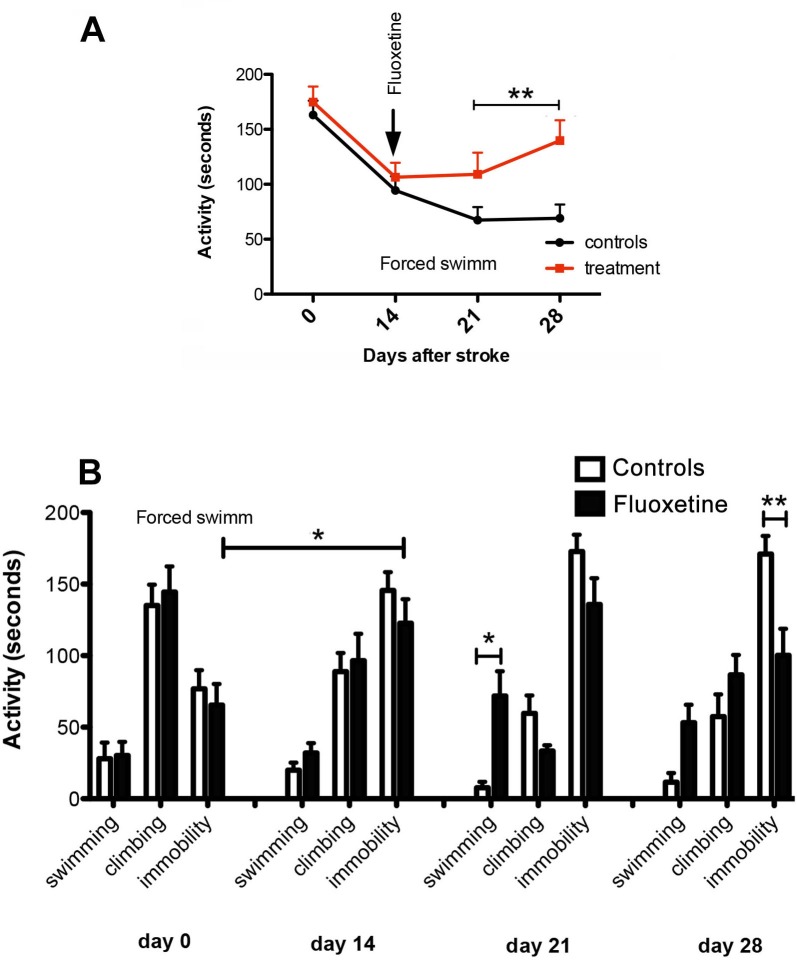
Assessment of post-stroke depression in aged animals **A.** After stroke, forced swimm cumulative activity decreased steadily form day 1 to day 28. Fluoxetine treatment, however, reversed partially the cumulative activity (dashed line). **B.** By behaviour activity type, swimming had a modest contribution to the total activity index. Stroke led to an impairment of climbing activity that reached the lowest value at day 21 post-stroke while immobility increased steadily from pre-surgery levels to day 21. The anti-depressant treatment led to an improvement in the swimming behaviour mostly (5-fold increase, *p* = 0.001) followed by an improvement in immobility behaviour (1.8-fold, *p* = 0.02).

The forced swim test (FST) is commonly used for assessing antidepressant-like behavior in animal models. There are three predominant behaviors in the modified FST: immobility, swimming and climbing. The effects of lesion (stroke) and anti-depressant treatment with fluoxetine are shown in Figure 2. Stroke produced a decrease in cumulative activity (i.e. composed of swimming, climbing and immobility times) that decreased steadily from day 1 to day 28 (Figure 2A). Fluoxetine treatment, however, reversed partially the cumulative activity (Figure 2A, red line). When analyzed by activity type, swimming itself had a modest contribution to the total activity index. As expected, stroke led to an impairment of climbing activity that reached the lowest value at day 21 post-stroke. Likewise, immobility has increased steadily from pre-surgery levels to day 21. The anti-depressant treatment led to an improvement in the swimming behavior mostly (5-fold increase, p=0.001) followed by an improvement in immobility (1.8-fold, p= 0.02) (Figure 2B).

### Age-specific post-stroke up-regulation of Htr2B mRNA in the peri-infarct area of rats

**Figure 3:**
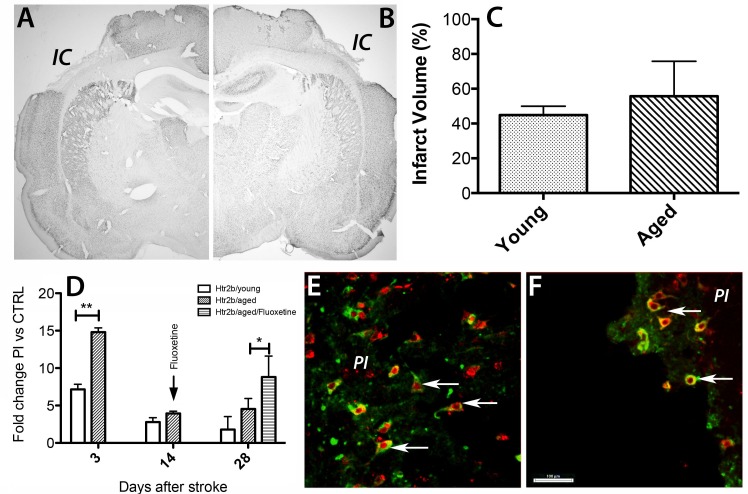
Age-specific post-stroke up-regulation of Htr2B mRNA in the peri-infarct area of rats **A.**-**C.** By day 14 the cortical infarcts were similar in size in both age groups i.e. 45% of total cortical volume in young rats and 55.7% in aged rats. **D.** By RT-PCR at day 3 post-stroke there was a 15-fold up-regulation in the expression of Htr2b mRNA that was 7-fold higher in the lesioned area of aged animals than in young animals (*N* = 7/age group). Fluoxetine treatment, however, caused by day 28, a significant increase (1.9-fold) in the Htr2b mRNA levels in the perilesional area of aged rats **E.**. In the perinfarcted area of aged rats HTR2B-positive cells (green) were co-localized with NeuN-positive nuclei (red) at day3 after stroke in aged rat brains (arrows). **F.** Although the mRNA expression was largely down-regulated by day14 post-stroke, the HTR2B/NeuN co-localization persisted in the peri-infarcted area of aged rats (arrows).

Immunohistochemistry with NeuN, a marker of neuronal nuclei was used to reveal details of the infarcted area [27]. Although the development of the infarct was more rapid in aged rats in the first 3 days after stroke [31] by day 14 the cortical infarcts were similar in size in both age groups i.e. 45% of total cortical volume in young rats and 55.7% in aged rats (Figure 3A-3C).

Of the genes possibly involved in post-stroke depression, we found by transcriptomics analysis using the Affymetrix platform, that the transcripts encoding the serotonin receptor 2B (Htr2b) but not Htr2a were upregulated after stroke in the aged rat stroke model. The expression also displayed a strong age effect, being more strongly upregulated at day 3 in aged than in young rats. The Htr2b mRNA levels returned almost to the pre-stroke level by day 14 [25]. To validate these results, we performed RT-qPCR. At day 3 post-strokewe found by RT-qPCR,a 15-fold up-regulation in the expression of Htr2b mRNA, that was 7-fold higher in the lesioned area of aged animalsthan in young animals (Figure 3D). Ht2b mRNA levels decreased thereafter in the perilesional area in both age groups. Fluoxetine treatment, however, caused, by day 28, a significant increase (1.9-fold) in the Htr2b mRNA levels in the perilesional area of aged rats (Figure 3D).

To determine the phenotype of Htr2B-positive cells, we performed double-immunofluorescence staining using a mouse antibody against HTR2B (green) and a rabbit antibody raised against NeuN (red). We found that HTR2B-positive cells were co-localized with NeuN-positive nuclei at day3 after stroke in aged rat brains (Figure 3E, arrows). Although the mRNA expression was largely down-regulated by day 14 post-stroke, the HTR2B/NeuN protein co-localization persisted in the peri-infarcted area (Figure 3F, arrows).

### HTR2B is expressed by neurons and astrocytes in the peri-infarcted area of human subjects

**Figure 4:**
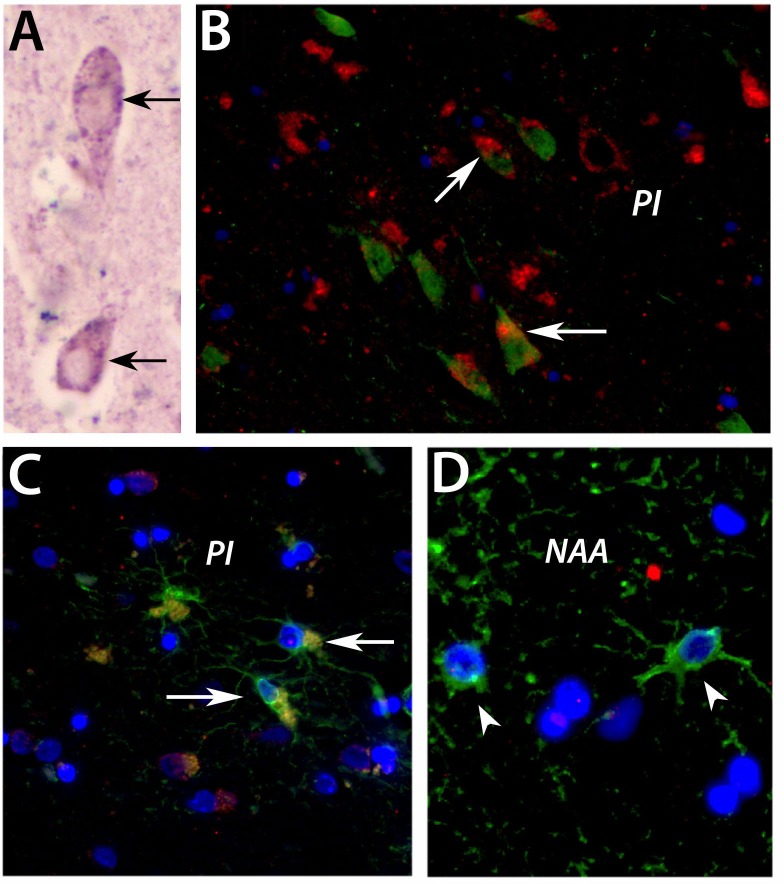
Histochemical localization of HTRB2B in the peri-infarct area of human subjects By chemical immunostaining, the HTR2B receptor in human subjects has a perinuclear localization (**A.**, arrows; Vector blue-grey development) in the penumbral region of post-mortem stroke patients. By immunofluorescence, a similar co-localization was observed in the peri-infarcted area between HTR2B-positive cells (green) and cells labeled by beta III tubulin (red), a marker of mature neuronal cells (**B.**, arrows). In addition to the neuronal co-localization, we also noted the co-localization with astrocytes labeled with FITC-GFAP (**C.**, arrows) in the penumbral region of post-mortem stroke patients. However, there was no co-localization with astrocytes in the unlesioned, contralateral region of the same post-stroke patient (**D.**, arrowheads). Bar, 100 μm. *Abbreviations*: PI, periinfarct.

By chemical immunostaining, the HTR2B receptor in human subjects has a perinuclear localization in the penumbral region of post-mortem stroke patients (Figure 4A, arrows). By immunofluorescence, a similar co-localization was observed in the peri-infarcted area between HTR2B-positive cells (green) and cells labeled by beta III tubulin (red), a marker of mature neuronal cells (Figure 4B, arrows). In addition to the neuronal co-localization, we also noted the co-localization with astrocytes labeled with FITC-GFAP (Figure 4C, arrows) in the penumbral region of post-mortem stroke patients. However, there was no co-localization with astrocytes in the unlesioned, contralateral region of the same post-stroke patient (Figure 4D, arrowheads).

### HTR2B is surrounded by activated microglia in the peri-infarcted area of aged rats and human subjects

**Figure 5:**
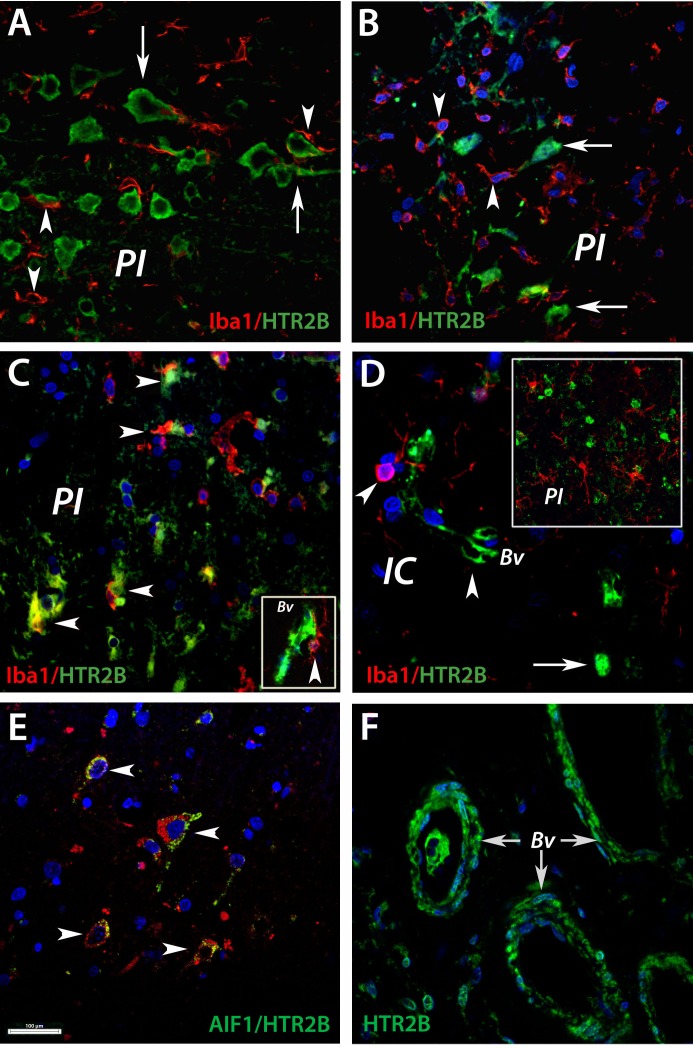
HTR2B is co-localized with brain macrophages in the perinfarcted area of aged rats and human subjects **A.** In the peri-infarcted area of young animals, activated microglia (arrowheads) was seen in close association with HTR2B^+^ neurons (arrows). The close proximity of HTR2B and brain microglia was less evident at day 14 post-stroke **B.**. **C.** In aged rats, numerous microglial cells immunopositive for IbaI were wrapped around neurons expressing HTR2B (arrowheads), suggesting a pre-phagocytic pathology. In addition, a robust co-localization of the microglia marker Iba1 with HTR2B-expressing blood vessels (inset; Bv). **D.** At day14, the co-localization between Iba1 and HtR2B was less evident in aged animals. **E.** In the peri-lesional area of human stroke victims we noted numerous phagocytic microglia expressing AIF1 (red), co-localized with HTR2B-expressing neurons (green). **F.** In remote areas with reference to the infarct core, numerous blood vessels were positive for HTR2B (green, arrows). Bar, 100 μm. *Abbreviations*: Bv, blood vessel; IC, infarct core; PI, periinfarct.

To gain insight into the cellular function of the protein, we further performed double-immunofluorescence staining for HTR2B and Iba1, a marker of brain microglia. In the peri-infarcted area of young animals activated microglia (Figure 5A, arrowheads) was seen in close association with HTR2B^+^ neurons (Figure 5A, arrows). The close proximity of HTR2B and brain microglia was less evident at day 14 post-stroke (Figure 5B). In aged rats, however, a co-localization of the microglia marker Iba1 with HTR2B-expressing neurons and blood vessels (Bv) (Figure 5C) was seen at day 3 post stroke. Despite persistent expression of Iba1 in the peri-infarcted territory (Figure 5D, inset), the co-localization between Iba1 and HtR2B was less evident by day 14 after stroke in aged animals (Figure 5D).

In the peri-lesional area of human stroke victims we noted numerous phagocytic microglia expressing AIF1 (Figure 5E, red), co-localized with HTR2B-expressing neurons (Figure 5E, green). Furthermore, numerous blood vessels were positive for HTR2B (Figure 5F, green, arrows) in remote areas with reference to the infarct core.

### Chronic fluoxetine treatment stimulates post-stroke neurogenesis in aged animals

**Figure 6:**
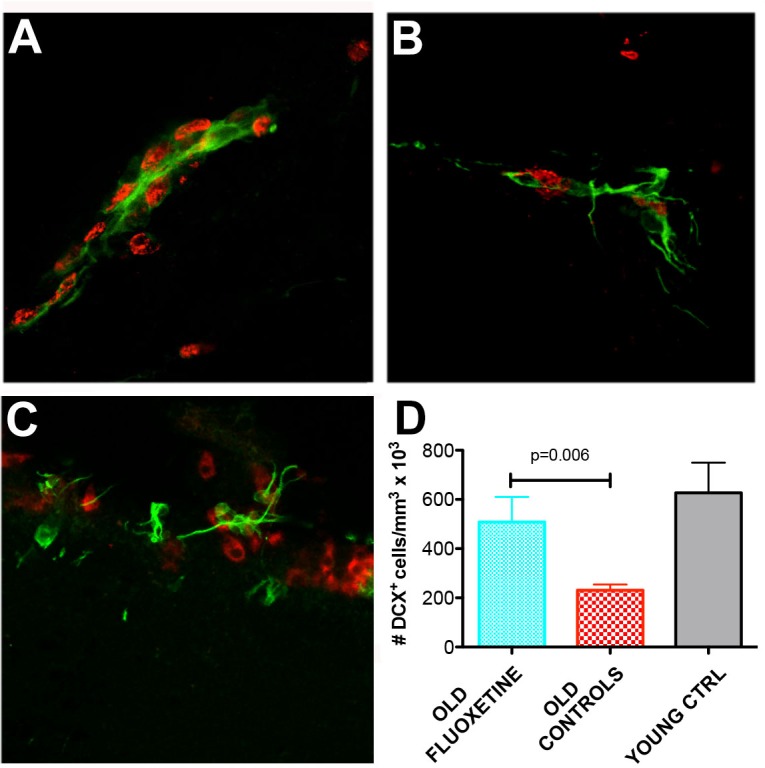
Fluoxetine stimulates post-stroke neurogenesis in aged animals **A.**,**D.** Note the vigorous post-stroke neurogenesis as evidenced by an increased number of DCX^+^ cells in the aged brains of animals treated with fluoxetine (2.2-fold, *p* = 0.02) as compared to the modest expression in the brains of control animals **B.** so that the number of DCX^+^ in treated animals was almost similar to the number of DCX^+^ found in the subventricular zone (SVZ) of young animals ipsilateral to stroke **C.**.

Fluoxetine is an antidepressant of the selective serotonin reuptake inhibitor (SSRI) family that has a recognised clinical efficacy and safety profile [33]. Since the underlying mechanism may involve neurogenesis [34], we investigated the immunoreactivity of the early neuronal marker doublecortin by immunofluorescence in the lateral ventricle region of aged rats after stroke. We noted vigorous post-stroke neurogenesis as evidenced by an increased number (2.2-fold, p = 0.02) of DCX^+^ cells in the aged brains of animals treated with fluoxetine as compared to the modest expression in the brains of control animals (Figure 6A vs 6B) so that the number of DCX^+^ in treated animals was almost similar to the number of DCX^+^ found in the subventricular zone (SVZ) of young animals ipsilateral to stroke (Figure 6C, 6D).

## DISCUSSION

Despite the fact that a high proportion of elderly stroke patients develop mood symptoms, there is, as yet, little insight into the underlying neurobiological mechanisms. Following stroke, aged animals exhibit a depressive behavior especially in the first week post-stroke. We found that the expression of serotonin receptor type B mRNA was markedly increased in the peri-lesional area of aged rats as compared to the younger counterparts. Further, histologically, HTR2B protein localisation was closely associated with degenerating neurons and activated microglia both in aged rats and human subjects.

### Aged rats after stroke display a markedly depressive behaviour

Following infarction, aged rats are refractory to movement and display a loss of appetite, eating and drinking a fraction of what young animals consume. Indeed, it has previously been demonstrated that aged animals lose some weight in the first week following stroke [26]. Our previous data showed that this was at least partially attributable to the surgical procedure itself during the first 3 days [26, 27, 35], but thereafter it was obviously due to a depressive behaviour manifested by the lack of interest in sucrose consumption and an increase in the immobility time in the forced swim test. This depressive behaviour could be due to differentiation of serotoninergic neurons in the grey matter [36]. Generally, there is a abnormal 5-HT neurotransmission with increasing age that contributes to the deterioration of cognitive processes in ageing, including depression [37]. It is well known that young animals recover readily form stroke. In fact, aged animals exibit a delay of several days in their recovey [26]. In addition to increased cortical damage in aged animals, we believe that young animals simply have better homeostatic mechanisms allowing for behavioral recovery by functional compensation. However, as we and others previously described, this behaviour improves abruptly upon exposure to an enriched environment [26, 33, 39]. Conversely, keeping the post-stroke aged rats in isolation exacerbated the depressive behaviour [26] suggesting that depression may be at the core of decreased motility and feeding and apathic behaviour [38, 39]. Indeed, earlier studies have implicated 5-HT2BRs in anxiety, schizophrenia, autism, migraine, and depression [40, 41].

### Identification of an age-related increase in Htr2B mRNA after stroke

The pattern of brain distribution suggests that the activation of 5-HT2 receptor subtypes may be implicated in the regulation of mood disorders [42]. Indeed we found that Htr2B mRNA but not Htr2A mRNA, was robustly increased in the perilesional frontal area of aged rats suggesting that the interruption in the serotoninergic innervation of cortical neurons by stroke may lead to an increased expression of Htr2B mRNA and serotonin receptors in the motor and frontal cortex.

Despite the wide occurence in the brain, the function of the 5-Htr2B receptor is not precisely known. In the brain stem, the 5-HT2B receptor modulates the respiratory network discharge properties [40] and within the vascular system, Ht2B receptors are involved in the regulation of the smooth muscle tone [43]. Moreover, because the available ligands do not discriminate between type B and type C receptors, it may be that many functions attributed to the type C receptor could have been fulfilled by the type B receptor as well [44].

Among the few studies directly addressing the function of the Ht2B receptor, it was shown that the Htr2B receptor agonist alpha-methyl-5-(2-thienylmethoxy)-1H-indole-3-ethanamine (BW723C86), increased food consumption and elicited anxiolysis in a social interaction model [45]. More recently, studies in Htr2B KO mice have shown that the Ht2B receptor is required for the hyperlocomotion induced by the “club drug” 3,4-methylenedioxymethamphetamine (MDMA; also known as ecstasy) and modulate the release of serotonin [5-hydroxytryptamine (5-HT)] stores from nerve terminals [46, 47].

Cognitive impairment and depression are the most commonly reported neuropsychiatric symptoms, both occurring in approximately one third of patients after stroke [48]. Apathy is most commonly defined as a syndrome of diminished goal-directed behavior, emotion, and cognition [49]. This occurs in a third of patients after stroke as an independent syndrome, although it may also occur as a symptom of depression or dementia [50, 51]. “Apathetic” PSD, associated with bilateral basal ganglia damage, and “affective” PSD, associated to left frontal lobe lesions [52]. Moreover, a relationship between apathy and hypoperfusion of both frontal lobes after stroke has reported [53, 54]. Since the behavior of our aged rodents was more „apathetic“ we hypothesize that the increased HT2B mRNA and protein expression in neurons around the peri-lesional area was due to the interruption of asceding inputs from basal ganglia which normally supply neurons in the frontal cortex with biogenic amines like 5-HT and dopamine. Our hypothesis is supported by animal studies reporting an upregulation of Htr2B mRNA and protein in response to the serotonergic deafferentation to compensate for the loss of serotonin input [55] and several clinical studies in elderly patients that reported an association between old age and apathy in stroke survivors [42, 56, 57] and normal elderly subjects [51]. Furthermore, apathy is significantly more represented in late onset depression than in early onset depression [58].

Previous studies have described that the selective serotonin reuptake inhibitors may have a beneficial effect in PSD therapy, but the role of SSRI treatment after stroke in aged animals has not been studied. Indeed, fluoxetine treatment alleviated the effect of stroke on the anhedonic behaviour indicated by an improvement in the swimming and immobility behaviour and redressed, to some extent, the expression of the Hrt2B mRNA. Our results are supported by recent suggestion that serotonin receptors are required for fluoxetine to exert its action. Thus, deletion of serotonin 1A receptor from the dentate gyrus in the hippocampus abolished the anti-depressant activity of the drug [59]. In addition, we wanted to exploit the anti-inflammatory effect of fluoxetine during the subacute phase in aged animals [60].

Increased cell proliferation and neurogenesis in response to chronic but not acute fluoxetine treatment may be one mechanism underlying the anti-depressant activity of fluoxetine [61]. This mechanism may also occur in the aged rats. Indeed, recent work has shown that chronic post-stroke treatment with fluoxetine increased the number of neuroblasts both in the SVZ and hippocampus of aged Wistar rats. However, since fluoxetine treatment did not influence either the survival of newly generated cells nor the performance in sensorimotor recovery [62] it is not currently known whether the newly generated neurons are functional *in vivo*. It has been reported that such neurons may be functionally active in hippocampal slices, a model very different from an *in vivo* situation [63].

Whether stroke stimulates endogenous neurogenesis is still debated especially in aged subjects where neurogenesis is normally decreased [64-68]. Recent data indicate that the action of fluoxetine on neurogenesis is highly dependent on the age of the treated individual [69]. Although several studies suggest no significant influences of fluoxetine on neurogenesis in the SVZ [61, 70] of experimental animals, it has been shown that stroke per se greatly increases neurogenesis in the subventricular zone[71]. Moreover, neurogenesis in the subventricular zone and nearby striatum after stroke is similar in young and old animals, indicating that this potential mechanism for self-repair also operates in the aged brain [72]. In addition, the beneficial effects of fluoxetine could be also due to the pleiotropic anti-inflammatory effect [60, 73]. Nevertheless, fluoxetine did stimulate post-stroke neurogenesis in the SVZ of aged animals and was associated with an improved anhedonic behavior and an increased activity in the forced swim test. However, whether endogenous neurogenesis contributes to an improved hedonic behavior has still to be established.

### Study limitations

The variability of injury size and injury location following MCAO model is considerably high; (ii) extent of fluoxetine treatment beyond 14 days; (iii) since fluoxetine may also have an anti-inflammatory effect during the subacute phase in aged animals, it is not clear in what ways this effect may have impacted post-stroke neurogenesis; (iv) mechanistically, we cannot clearly distinguish between post-stroke depression and apathy.

## CONCLUSIONS

Because of the lack of a precise function of the HT2B receptor in the brain, we can only speculate about the functional significance of the massive upregulation of this receptor after stroke in the aged brains. A similar upregulation of HT2B receptors has been recently reported for amyotrophic lateral sclerosis (ALS), a fatal neurodegenerative disorder affecting upper and lower motor neurons [67]. Loss of serotoninergic transmission led to a compensatory constitutive upregulation of Htr2B receptors on motor neurons causing an intrinsic hyperexcitability and subsequent spasticity [55]. A prolonged neuronal hyperexcitability may also lead to degeneration of serotoninergic neurons, and consequently attract activated microglia as we consistently observed this in the lesioned area of aged animals and autopsy samples of stroke patients. Finally, treatment with fluoxetine stimulated post-stroke neurogenesis in the SVZ and was associated with an improved anhedonic behavior and an increased activity in the forced swim test in aged animals.

## MATERIAL AND METHODS

### Animals

30 young (3 to 4 mo of age) and 46 aged (19 to 20 mo) male Sprague-Dawley rats, bred in-house, were used. Body weights ranged from 290 to 360 g for the young rats and from 520 to 600 g for the aged rats. After behavioural testing, the two age groups were divided randomly into 3-, 14- and 28 day post-stroke survival groups. The group sizes for the aged rats were larger to compensate for the higher post-ischemic mortality rate. After 14 days post-stroke the aged group was further divided into a treatment group (N = 15) and a control group (N = 13). The numbers reported in the results refer to the number of animals that survived the surgery and completed the 4-week testing period. The rats were kept in standard cages in a temperature (22°C), humidity (40–60%), and light period (07.00–19.00h) controlled environment and had free access to food and water. The Animal Experimentation Institutional Animal Care and Use Committee of the University of Medicine and Pharmacy Craiova as meeting the ethical requirements of the EU legislation on the protection of animals used for experimental purposes approved all experiments.

### Reversible occlusion of the middle cerebral artery

Eighteen hours prior to surgery, rats were deprived of food to minimize variability in ischemic damage that can result from varying plasma glucose levels [28]. Water remained available at all times. In all cases, craniotomy was performed between 9:00 and 13:00. Animals from the control and experimental groups were randomly subjected to cerebral infarction that was induced by the focal interruption of blood flow by transiently lifting the middle cerebral artery with a tungsten hook, as previously described [27]. To prevent continued perfusion of the target region by arterial collaterals, both common carotid arteries were then occluded by tightening pre-positioned thread loops. Throughout surgery, anaesthesia was maintained by spontaneous inhalation of 1-1.5% isoflurane in a mixture of 75% nitrous oxide and 25% oxygen. Body temperature was maintained at 37°C by a Homeothermic Blanket System (Harvard Apparatus) and the tail artery was catheterized to allow the continuous measurement of blood pressure and the withdrawal of blood samples for determination of pH and blood gases (Blutgassystem IL 1620, Instrumentation Laboratory, Munich), as well as arterial glucose levels (Omnican7 Balance, B. Braun, Melsungen). Local changes in blood flow were monitored using a laser Doppler device fixed in place using a tiny cylinder nearby the occluded artery (Perimed, Stockholm, Sweden), and blood gases were measured at several timepoints during ischemia. A decrease in the laser Doppler signal to <20% of control values was considered to indicate successful MCA occlusion. After 90 minutes, the hook was released and the common carotid arteries were re-opened.

### Treatments

Aged rats were given a fine suspension of fluoxetine daily, an anti-depressant drug, in saline (10 mg/Kg body weight) administered intraperitoneally at day 14 after stroke and was continued for two weeks. The control group was treated with vehicle (saline). To label newly generated cells, all rats were treated with once-daily i.p. injections of bromodeoxyuridine (BrdU; 50mg/kg body weight; Sigma, Germany) during the first week post-stroke, continued at alternating days thereafter for a period of 14 days in total.

### Behavioural tests

The testing procedure involved two experimenters, one who performed the surgery and was in charge of handling the animals according to group assignment, and another who carried out the behavioural tests and who was not aware of the animals' group assignments.

### Forced swim

The forced swim test was used to assess the time of active/passive behavior when animals are placed in an inescapable cylinder with water (50 cm water depth) for 5 min. Time sampling technique was used to rate the predominant behavior over a 60-s interval. Active behavior was analyzed by three different form of active behavior: (1) climbing behavior- upward-directed movements of the forepaws along the side of the swim chamber; (2) swimming behavior; and (3) immobility, determined as absence of any directed movements of animals' head and body was scored during the first 2 min of the test [29].

### Hedonic behavior

Sucrose consumption during a period of 24 hours was used as a measure of hedonic behavior as described in detail previously [30]. Briefly, 4 days before surgery rats were given, for 24h, a free choice between two bottles, one filled with 2% sucrose solution, the other containing tap water. After a break of two days, the rats were primed again with 2% sucrose for 24 hrs. To control for potential effects of side preference, the position of the bottles was switched after 12 h. In order to balance the air temperature between the room and the drinking bottles, they were kept in the same room where the testing took place. In order to decrease stroke variability due to sucrose consumption, one day before surgery rats were given tap water. After the MCAO was performed, spontaneous activity and hedonic behaviour (sucrose and water levels) were monitored every seventh day and recorded for 4 weeks (Figure 1A). Throughout the experiment the two bottles (water and sucrose) were switched once per day, to prevent possible effects of side preference in drinking behavior. Consumption of liquids was evaluated by weighing the bottles before and after the test. Sucrose intake was given as the amount of consumed sucrose in mg per gram body weight [30].

### Tissue, RNA and protein analysis

Subsequent to a survival time of 3-, 14- and 28 days, the rats were deeply anesthetized and perfused with buffered saline followed by buffered, 4% freshly depolymerized paraformaldehyde. The brain was removed, post-fixed in buffered 4% paraformaldehyde for 24 h, cryoprotected in 20% sucrose prepared in 10 mmol/L phosphate-buffered saline, flash-frozen in isopentane and stored at −70°C until sectioning. For total RNA and protein isolation, the rats (N = 7) were perfused with buffered saline only, and the brain was cut into 2 mm slices that were dipped in 2% 2,3,5-triphenyltetrozolium chloride (TTC) solution so that the infarct core could be visualized and microdissected under a dissecting microscope as previously described [25].

### Real Time Quantitative PCR

Total RNA was isolated using TRIzol reagent (Invitrogen Life Technologies, Germany), as described by the manufacturer, followed by DNase 1 (Ambion) digestion and further purification using the RNeasy Mini extraction kit (Qiagen, Hilden, Germany). Purified total RNA was used for cDNA array assay and real-time PCR quantification.

For real-time PCR, 2μg of total RNA was reverse-transcribed using random hexamers and the reverse transcription reagents supplied by Life Technologies (Karlsruhe, Germany). The PCR reaction was set up by mixing 10ng of cDNA, primers, and Master mix (PeqLab, Germany), and real-time PCR amplification was performed as follows: one cycle of 15min at 95°C and 45 cycles in three steps each (95°C for 30-s, 58°C for 30-s, 72°C for 30-s) using a real-time PCR cycler (MyiQ Cycler Bio-Rad). A standard curve was generated by plotting the log_10_ [target dilution] of template on the X-axis against the Ct value from serial dilutions of target DNA on the Y-axis. The efficiency of PCR amplification was 98%. The relative expression level of *Htr2B* and Rpl13a (as the housekeeping gene) was determined based on the standard curve equation generated for each individual gene.

### Immunohistochemistry

Sections (25 μm-thick) were cut on a freezing microtome and processed for immunohistochemistry as free-floating material using an automatic staining device (www.tingomat.com) capable of staining several hundreds of sections simultaneously. Briefly, after incubation with blocking solutions containing 3% donkey serum/10 mmol/L PBS/0.3% Tween 20, tissue sections were exposed overnight at 4°C to mouse anti-NeuN (1:1000, Millipore, Germany) diluted in PBS containing 3% normal donkey serum and 0.3% Tween 20. After extensive washing in PBS containing 0.3% Tween, sections were incubated overnight at 4°C with biotinylated donkey anti-mouse IgG (Jackson ImmunoResearch Laboratories, West Grove, PA) diluted 1:4000 in PBS containing 1% normal donkey serum and 0.3% Tween 20. After washing in PBS, sections were incubated for 4 hrs at room temperature in ABC Elite reagent (Vectastain Elite Kit, Vector) diluted 1:100 in PBS containing 0.3% Tween 20. The antibody complex was then visualized with 0.025% 3′,3′ diaminobenzidine (DAB) and 0.005% hydrogen peroxide in 100 mmol/L Tris buffer (pH 7.5).

### Determination of infarct volume

To assess the size of the infarct induced by transient focal ischemia, every tenth section was stained NeuN as previously described [31]. Briefly, images of the stained sections were taken to cover the entire infarcted area, which was then calculated as the sum of partial areas using Image J. Integration of the resulting partial volumes gave the total volume of the ipsilateral hemisphere along with the total volume of the cortical infarct; lesion volume was then expressed as percent of the hemispheric volume.

### Doublecortin-BrdU double staining

Cryostat, free-floating sections of 25μm were fixed in 4% paraformaldehyde for 15 minutes and then washed extensively with PBS After incubation in 50% Formamide/2X SSC for 2h at 60°C, sections were washed again, first in 2x SSC and then in 10x PBS. After denaturation in 2N HCL at 37°C for 40 minutes, sections were made neutral by adding 0.1M Borate buffer (pH 8,5). Thereafter sections were incubated sequentially with the guinea pig anti-doublecortin (1:2000; Millipore, Germany) antibody overnight at 4°C followed by donkey anti-guinea pig IgG-biotin (Dianova, Hamburg, Germany) and streptavidin Alexa 488 (Life Technologies, Karslruhe, Germany). Finally sections were incubated with rat anti-BrdU antibody (1:2000, AbD Serotec, Puchheim, Germany). BrdU-positive cells were visualized by incubating with Cy3-conjugated donkey anti-rat IgG (H+L) (1:3000, Dianova, Hamburg, Germany).

### Immunofluorescence on rat brain sections

Sections (25 μm-thick) were cut on a freezing microtome and processed for immunohistochemistry as free-floating material using an automatic staining device (www.tingomat.com). For phenotyping, rat sections were blocked and incubated overnight at 4°C either with rabbit anti-NeuN (1:1000, Millipore, Germany) or anti-GFAP (1:1000, Abcam) or rabbit anti Iba1 (1:3000, WAKO Chemie, Germany) diluted in 3% goat serum, 1x PBS and 0,2% Tween. After overnight incubation at 4°C, sections were washed and incubated with the secondary antibody, goat anti-rabbit-CFL 555 (1:1000 dilution, Santa Cruz, Heidelberg, Germany). After a short fixation in 4% paraformaldehyde, serotonin receptor 2B-expressing cells were visualized by incubating sequentially with mouse anti-HTR2B (1:2000, Pharmingen) followed by goat anti-mouse HRP polymer (1:10 in 1% goat serum; Histofine, Nichirei Technology). Finally, cells were stained using tyramide-FITC. Then sections were mounted in ProLong® Gold Antifade reagent with Hoechst 33342 (Life Technologies, Karlsruhe, Germany) to stain nuclei.

### Immunofluorescence on human brain sections

To analyze HTR2B expression after ischemic stroke, formalin-fixed, paraffin-embedded archived brain tissue blocks containing both cortical lesional and peri-lesional areas were selected from 12 stroke patients with a survival post-stroke time ranging from 12 h to 7 days. Death was attributed to complications such as massive edema and brain herniation with rostrocaudal deterioration, or cardiovascular arrhythmias. Written informed consent to autopsy was obtained for each patient from the relatives or their caregivers. Demographic data of patients and controls are given in Table 1.

**Table 1:** Case demographics for stroke patients

Pacient	Age	Sex	Survival time	Stroke type	Region analyzed
1	78	F	6 d	IS/ICH	CA
2	51	F	36 hrs	IS	CA
3	68	M	12 hrs	IS	CA
4	67	M	40 hrs	IS	CA
5	51	F	36 hrs	IS	CA
6	NA	M	20 hrs	IS	CA
7	66	M	30 hrs	IS	CA
8	72	M	8 d	IS/SAH	CA
9	78	M	18d	IS	CA
10	88	F	7d	IS/SAH	CA
11	68	M	27d	IS	CA

IS indicates ischemic stroke; ICH, intracerebral hemorrhage;

SAH, subarachnoid hemorrhage; CA, cortical area

7μm sections were cut and mounted on positively charged slides (Superfrost®), then dewaxed and rehydrated. Antigen retrieval was performed in heated 1mM citric acid (pH6) and non-specific antigen binding was blocked in 10% goat serum in PBS. Sections were exposed to anti-5-HT2BR antibody (1:50, BD Bioscience) and either anti-AIF1 antibody (1:100, Abcam) or anti-beta III Tubulin antibody (1:1000, Abcam) diluted in 1% goat serum in PBS, then detected using Alexa Fluor 488 and 594 secondary antibodies (Life Technologies). Sections were mounted in ProLong® Gold Antifade reagent with DAPI (Life Technologies) and visualized on a Leica DM6000 fluorescent microscope.

### Microscopy

Confocal microscopy images were acquired using a Zeiss LSM710 laser-scanning confocal system with spectral detection capabilities, and Zen 2010 software version 6.0 (Carl Zeiss Microscopy GmbH, Jena, Germany) was used for image acquisition and analysis. Excitation light was provided by 488, 543, and 634 nm laser lines; fluorescence emission was detected at 500-530 nm for FITC (green), 550-600 nm for CFL 555 (red) and 360-486 nm for Hoechst (blue) in separate tracks, using a confocal aperture of 1 Airy unit. Some of the images were acquired as z-stacks and 3D reconstruction was performed.

### Cell Quantitation

A quantitative estimate of the number of DCX- and BrdU-immunopositive cells was obtained by counting the cells in 25-μm-thick sections in volume units measuring 250μm × 250μm × 10 μm, employing a ‘random-systematic’ protocol (random start point for a systematic series of every 10th section through the infarcted volume) as previously described [5]. The area occupied by cells of interest was 30% of the total stained area. The relative mean number of co-localized cells was then calculated per group, time point, and age by multiplying the number of cells per section times 3.3 (the counting boxes that were quantitated covered one third of the area of each section) times the section interval of 10. Because co-localization in one confocal plane sometimes may be misleading as to the number of cells co-localizing, we counted double-labeled cells by 3-D reconstruction as described above. By rotating the 3-D image, we were able to determine precisely the number of co-localized cells [5].

### Statistical analysis

The age-dependency of anhedonia and the effect of treatment on the hedonic behavior were analyzed using the Mann Whitney test. The main effect of treatment on sucrose and total liquid consumption, forced swim, including swimming, climbing and immobility, was evaluated by using two-way ANOVA. Cell quantification was performed using the t-test. The level of significance (two-tailed threshold) was set at p ≤ 0.05. Data are presented as mean ± SD for all other data, as indicated for each figure.
